# LoRa-Based Data Mule Technology for Fuel Station Monitoring in Underground Mining

**DOI:** 10.3390/s26082369

**Published:** 2026-04-12

**Authors:** Marius Theissen, Qigang Wang, Amir Kianfar, Elisabeth Clausen

**Affiliations:** Institute for Advanced Mining Technologies (AMT), RWTH Aachen University, 52062 Aachen, Germany; qwang@amt.rwth-aachen.de (Q.W.); akianfar@amt.rwth-aachen.de (A.K.);

**Keywords:** LoRa, underground, mining, room and pillar, network, data mule

## Abstract

Digital mining has become a tangible reality in recent years and the digital revolution enables and requires data exchange for autonomous machines and operational flow management. LoRa technology and its underground propagation behavior can make an important contribution to this digitalization. This paper presents a Data Mule approach that enabled progress in digitalization at refueling stations in active underground mining areas of a mine near Werra, Germany, operated by the K+S Group. This demonstration aimed to automate manual data collection at fuel gauges by using a dynamic LoRa network. We used specially developed LoRa Data Mule modules for operations over many square kilometers. LoRa was chosen for its industrial functionality and long-range capabilities, particularly in underground environments. The Data Mule modules used were in-house-designed units with underground mining-rated casing and connectors, as well as commercial LoRa boards and custom communication protocols. Connectivity between all systems was realized at travel speeds of 20 to 40 km/h, with connection data successfully relayed for 180 to 770 m, despite 90° turns and no line of sight. It was shown that the LoRa Data Mule approach can be used in a network of remote but active data generation points.

## 1. Introduction

The mining sector has always integrated emerging technologies to enhance efficiency and safety, minimize environmental impact and reduce operational expenses.

It is becoming increasingly clear that digitalization is a trend driven by emerging challenges. These include declining ore quality, complex deposit structures, deeper mining operations and fluctuating market prices.

The overarching goal of this transformation is to develop an interconnected, autonomous mining environment that leverages real-time data for informed decision-making [1,2].

Expanding into a use-case-oriented study, ref. [2] provides an overview of the communication options in this context of the digital mining transformation. In mining, IoT is most frequently implemented through remote operations and advanced automation efforts [3]. The aim is to gather status information on mobile machines and personnel [4], transmit emergency situational data [5] or monitor data, for example, on rock stability [6]. All these IoT use cases necessitate a stable and reliable communication system for the transmission or exchange of (real-time) data.

However, deploying wireless communication infrastructure is particularly challenging in underground mining. Problems include but are not limited to a lack of field-tested data for a number of theoretically viable but untested communication options. The physical and legal limits have been taken to their extremes by varying mining environments and operational constraints, as outlined in [7]. Each mining operation has a unique natural geological structure paired with its mining method, level of mechanization and automation, as well as the spatial scale and production level of the operation.

Several approaches and implementation methods have been developed over the years for network design and deployment in underground mining environments. The research community has pursued both theoretical modeling and practical testing of these networks in active mining settings. One of the previous publications that explicitly identified and framed wireless communication and sensor networks as a solution to challenges in underground mining is [8]. In this study, the authors highlighted the key performance indicators reliability, power consumption, interoperability, and scalability. Subsequent experimental approaches have built on this foundation. For instance, detailed measurements of LoRa propagation in underground gold mines were reported in [9], providing valuable empirical data for low-power wide-area network (LPWAN) design. Comprehensive compilations and comparative analyses of propagation models have been published, most notably [10], which have presented explicit calculations that accounted for the geometric characteristics of mine drifts.

A broad spectrum of communication technologies has been evaluated in various underground scenarios in order to uncover specific propagation effects and to validate existing predictive models. In this context, ref. [11] stands out for its investigation of 5G signal behavior in curved drifts, highlighting the feasibility of next-generation cellular systems.

Guided by these studies and efforts, several active-network applications have been conceived and prototyped. Static sensor networks, in particular, have been proposed which enhance safety in coal mines; representative contributions include [12,13,14,15]. In addition, wireless solutions for water-level monitoring and control have been demonstrated, for example in [16].

While the concept of Data Mules has gained traction in smart-city research [17,18] and previous studies have investigated underground wireless propagation, static sensor networks and individual communication technologies such as LoRa and 5G, the application of a LoRa-based Data Mule architecture for mobile refueling station monitoring in active underground mining has not yet been sufficiently addressed. In particular, existing work does not adequately cover scenarios with relocating operating points, NLoS conditions and the absence of permanent communication infrastructure. This research gap has motivated the present study.

This paper aims to determine the viability of a practical solution for a specific use case in an active underground room-and-pillar salt and potash mine operated by K+S near Werra, Germany. Within the NEXGEN SIMS project, which focuses on developing autonomous carbon-neutral mining processes [19], a need arose to automate manual data collection from fuel gauges accross an entire mining district covering several square kilometers. Several stipulations and requirements were defined by mine operation officials.

The communication system was required to operate without a permanent network infrastructure, as refueling stations advance alongside the excavation front. For a smooth and easy transition, no changes to work procedures and no replacement of existing machines and infrastructure were made.

Thus, a specialized approach was developed based on LoRa communication technology, alongside a Data Mule network structure around the fuel stations. LoRa, short for long range, is an emerging solution for sub-GHz communication. It forms a low-power, wide-area network (LPWAN), which operates in the unlicensed ISM bands for the internet of things [20]. These features give LoRa a unique position in the field of communication options, as it can tackle tasks across extensive areas with limited power grids at a reliable but low data rate. It has demonstrated its effectiveness in urban settings, in promising theoretical studies, and in behavioral studies in mining environments [9,21]. A groundbreaking work on the broader topic is the dissertation by T. Krichler [22].

The structure of this paper is as follows:

1. Introduction
2. Use-Case Description and Methodology
  - Overview of the refueling station systems and maintenance procedures.
  - Overview of LoRa and the Data Mule systems.
3. Hardware and Software Configuration
  - Description of the different hardware units forming the Data Mule network.
  - Layout of software architecture and system-wide implementation.
4. Results
  - LoRa coverage.
  - Physical and digital data paths.
5. Discussion
  - Viability of the approach.
  - Implications for future work flows.
6. Conclusions
  - Main results.
  - Outlook.

In summary, this study presents the concept, implementation and evaluation of a LoRa-based Data Mule architecture for monitoring refueling stations in an active underground mining environment. The main contributions of this paper are: demonstration of a LoRa-based Data Mule architecture for mobile refueling station monitoring in an active underground potash mine; practical integration of refueling stations, vehicles, and a server under NLoS conditions without permanent communication infrastructure; evaluation of communication feasibility at vehicle speeds of 20 to 40 km/h, with transmission distances up to 540 m; and discussion of applicability and limitations for comparable underground monitoring tasks.

## 2. Use Case Description and Methodology

In the use case LoRa Data Mule for Fuel Stations, a dedicated and dynamic network setup was demonstrated with the objective of creating automated and centralized data collection procedures. The goal was to find an alternative to manually sampling refueling stations, due to the associated costs and time consumption. Electricians manually recorded measurements at irregular intervals. These were then transferred to spreadsheets at different locations. This use case was addressed by researchers from the AMT RWTH Aachen Institute as part of the greater EU-funded NEXGEN SIMS project, with the aim of demonstrating dynamic network coverage for otherwise remote areas using LoRa.

The goals can therefore be divided into two categories; The first is scientific and technical: that LoRa propagation, supported by measurements from the potash mine, can provide accurate model-based predictions for real use cases. The second is cooperative: to test a novel network architecture combining LoRa and Data Mule in an environment where manual data collection workflows are currently used. This is an approach not previously implemented in such a setting.

Previous attempts to digitize fuel stations can be divided into two categories: underground and aboveground. Aboveground, 5G networks and LTE are already in use in fuel stations equipped with 5G sensors and many more applications [23,24,25], while in agriculture, LoRa technology is already used for remote-sensing applications such as measuring water levels and soil moisture [26,27]. Underground, direct connections between mobile sensor stations using these systems are still relatively new. Networks like 5G, designed for thousands of devices, represent a approach where a single network is tested for a high data, low latency use case. LoRa offers a unique combination of long range, autonomy, and low cost. Previous implementations have mostly been manual or involved testing over large and expensive networks like 5G or Wi-Fi [28,29,30]. These static networks are typically designed for automation tasks that require distinct operational procedures and budgets.

The location of the demonstration was the Werra potash mine operated by K+S, Germany. At a depth of around 800 m, the network and the refueling stations were linked in the active operating area.

The environmental conditions were comparable to those in the measurement series in [21]. In this measurement, the decisive factors are the drift shapes, the topology of the surfaces and Line-of-Sight (LoS) conditions.

### 2.1. Communication Requirements

The main requirement defined by the industrial partner was the absence of permanent infrastructure. The resulting system was required to follow the advancing excavation front and the NLoS nature of the environment should not impair or prevent its operation. The network was required to integrate seamlessly into daily operation, with vehicle speeds of up to 40 km/h, and any active machinery should not be exchanged but upgraded. This point also included user training and input. A completely autonomous and parallel network was required. With no new power grid extensions, a low-power network would be required. Any changes to the personnel routes was not possible, hence the network needed to operate in a drive-by mode, connecting spontaneously at any point in time.

**Figure 1:**
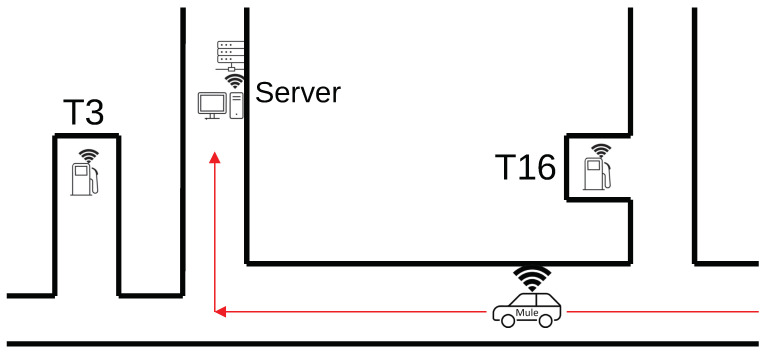
Schematic representation of a Data Mule round-trip flow envisioned to maximize usage of the regular routes taken by personnel for tasks unrelated to the refueling stations. T3 and T16 are refueling stations and the Server is collecting data from the Data Mule. The red arrows indicate the vehicle route.

LoRa-based network nodes were installed in special configurations on vehicles, server areas and several refueling stations with digital-level sensors, as seen in the schematic round trip in Figure 1.

Mine workers verified that the jointly agreed modifications to the power and signal connections of the vehicles, refueling stations and server location, which was a current transformer site, were securely and correctly installed. They undertook subsequent validation runs in the vehicles together with the AMT Institute research staff. This resulted in a network link-up within three seconds over approximately 100 m without LoS, with data comparison between units of 0.5 kB in extreme cases, with subsequent validation of the data. The aim was to enable this automatic exchange without staff intervention during normal operation.

**Figure 2:**
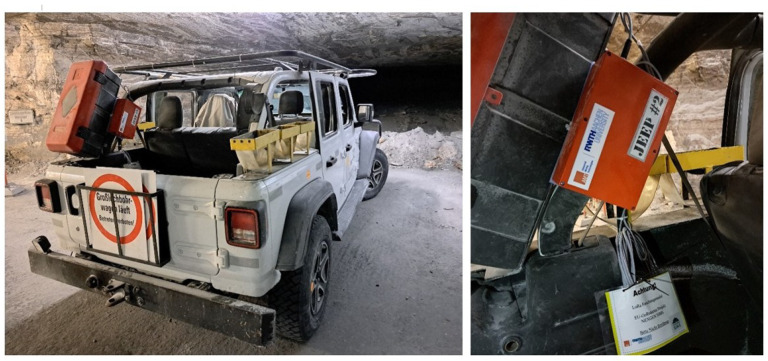
Upgraded Jeep. A Data Mule node was mounted outside of the steel frame to maximize signal strength. The power connection was run directly from the battery next to the engine.

**Figure 3:**
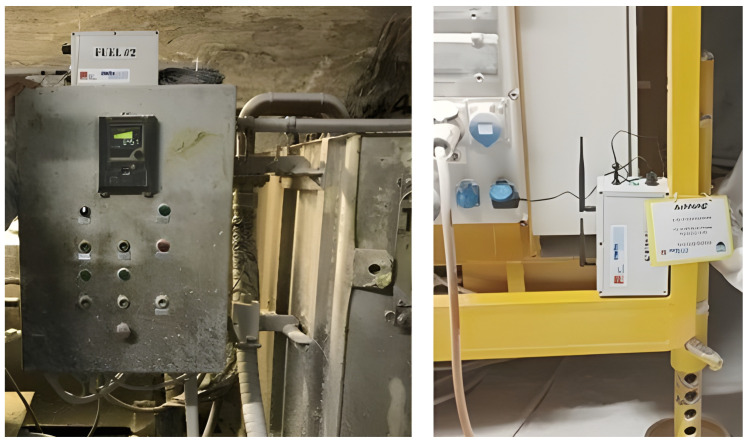
**Left**: LoRa node-upgraded refueling station. The unit was mounted outside the power supply box to ensure the best signal coverage. **Right**: Power supply unit connected to server LoRa box.

A key design objective was to eliminate manual detours by personnel to the refueling stations, which meant node connections had to be established spontaneously, even without LoS. As it was not possible to predict when the mules were within theoretical range of each other. Three different modules had to be developed for the mining environment, while accounting for dust, temperature and humidity, in order to realize the different node types, see Figure 2 and Figure 3. The planned network was based on knowledge of the special LoRa propagation found and investigated in the same mine but in a different area, as described in [21].

During the conceptualization phase of the LoRa Data Mule approach, a feasibility study was conducted [21]. As part of the hardware and software design considerations, models were developed within the NEXGEN SIMS project to provide initial predictions of LoRa propagation behavior underground. The so-called Relative Length Model, together with empirical data from measurements, served as a basis for estimating feasibility and validating network performance in underground environments [21]. According to both the model and the measurement series, an ideal range of approximately 500 m was expected on straight paths. Without this approach, most classical models like the family of dielectric models [31] or the fundamental Friis model would predict nearly 100% signal loss [32]. For a basic overview of potential models in the field of EM wave propagation see [33]. Due to the lack of other suitable models, especially for alternative network options, this work relied on measurement results and internally developed models to minimize the number of dependent variables in this measurement series.

It was assumed that the devices could continuously attempt to establish a connection prior to each successful transmission period, and external noise was negligible.

This use case reflects realistic operating conditions in underground mining, where reliable communication is critical for safety and automation. In these conditions, No Line-Of-Sight (NLoS) areas make it impractical to maintain a continuously connected network across all areas.

LoRa was chosen for this use case setup based on several factors: range, cost, and predictability of propagation in salt/potash mines. For comparison, the three major contenders for network options are 5G, Wi-Fi, and Bluetooth [2,34]. Candidates within smart IoT solutions include but are not limited to NFC, digital radio, Strizh, and Sigfox. Each of these options has its advantages and disadvantages, just like LoRa.

NFC offers only extremely short ranges [35], which could reduce manual logging but would still extend the personnel Jeep routes. Digital radio is an effective underground solution, especially for personnel communication; however, regulatory uncertainties exist. Digital radio is essential for communication between staff and ongoing operations. Attempting to address this layer only partially through constant traffic would be problematic and could not be implemented without significant arrangements.

Sigfox is a growing IoT solution [36], but it lacks the measurement data required to assess its applicability in underground mining for planning use cases. Wi-Fi is a serviceable option [37], but comes with higher upfront costs; thus, it currently functions mainly in fixed areas such as underground break rooms with permanently or partially wired sections. This setup does not align with the repositioning of the refueling stations.

Bluetooth is widely used in aboveground applications [38] but lacks sufficient signal budget to support a drive-by approach with Jeeps as required. Strizh is a Russian-based satellite communication service similar to StarLink [39,40]; however, this service has not yet been tested or deployed in German underground mines.

5G represents a centralized solution that is expanding for teleoperation [41] and automation underground [30]. However, deploying an extensive permanent 5G network for an initial single use case like refueling stations would be excessive. Still, implementation may be considered, alongside existing automation plans.

LoRa uniquely combines long-range capability, which was tested over several hundred meters underground [21], and low hardware requirements (see Section 3). To ensure the feasibility of the project, stable measurement data for communication options in underground potash mining was necessary. Therefore, LoRa data from a previous measurement series was analyzed [21]. A concise overview of four possible network approaches is shown in Table 1 in order to provide some structure to this theoretical list of points and characteristics.

### 2.2. Methodology and Setup

To evaluate the performance of a LoRa Underground Data Mule communication concept, a controlled experimental demonstration setup was designed. A structured methodology was applied to measure key performance metrics such as coverage, data rate and time taken to establish data transfer with passing vehicles under realistic constraints—see Table 2.

To further increase the transferability of the measurement results, operating resources in the active mining area were selected for the demonstration. Two refueling stations were designated, chosen to be as far apart as possible within the active zone. Additionally, a server with a constant power supply was set up, which was to be integrated into the local K+S data network in later versions of the setup. For the purpose of the demonstration, the server was operated separately from the K+S network and provided wireless access for data recovery. The vehicles used for daily electrical engineering operations were due to be upgraded—see Figure 2. These two Jeeps took routes past the filling stations and were equipped to act as Data Mules, with additional Mule-to-Mule communication capabilities. A dedicated protocol based on LoRa was used to provide communication between all nodes. Both the server and the refueling stations were located on drifts perpendicular to the main routes of the Data Mules, see Figure 3. All nodes, including those from the refueling stations and the server, were positioned in an NLoS configuration relative to all Data Mules. This arrangement posed significant challenges for network design, particularly concerning reliability and overall feasibility. Reliability is an issue with the constantly interrupted LoS. Most urban or suburban propagation models calculate the signal attenuation for the refueling station as insufficient [33]. In addition, reliability should not be influenced by human factors, i.e., no further checking of readings or targeted visits to refueling stations for inspection should be necessary, transmitting data only via LoRa and the network concept.

However, findings from the LoRa study by Theissen et al. [21] suggested that LoRa could serve as a viable communication technology even in NLoS scenarios. The measurements indicated that LoRa signals can propagate through underground drifts via reflective bouncing effects. A preliminary model of this propagation behavior is also presented in the referenced study and served as the conceptual basis for the present setup.

The nodes deployed at the refueling stations operated continuously in a publisher mode. In this configuration, a handshake protocol was broadcast at regular intervals over the standard European LoRa frequency band. This ensured that a Data Mule entering the communication range of a station triggers the data exchange process.

To enable this, the Data Mules were set up to continuously switch between subscriber mode, focused on the refueling stations, and publisher mode, linked towards the server node kept as a subscriber. This process was monitored live within the vehicle using an onboard interface. During the demonstration runs, both the data traffic and LoRa transmissions were recorded. The connections established between nodes were mapped in real time along with the position of the vehicle to visualize the effective reception zones.

**Figure 4:**
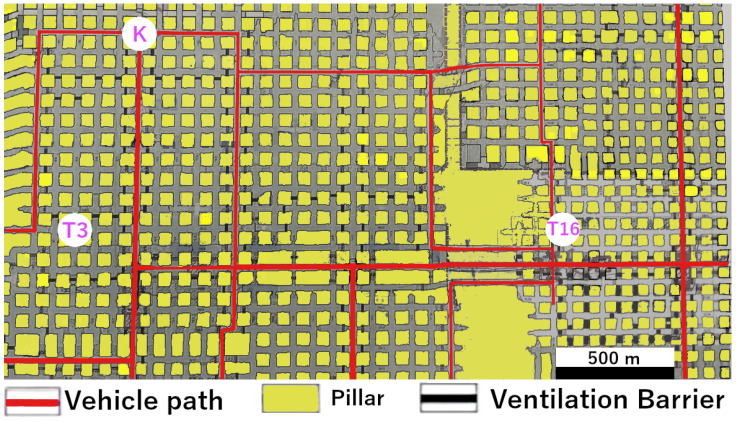
Excerpt from a map of the K+S mine near Werra, Germany. Points of interest like refueling stations and server areas are highlighted. T3 and T16 are refueling stations and K is the on-site label for the tipping point, expanded with the server unit.

Multiple runs were conducted at varying vehicle speeds, ranging from 20 km/h to 40 km/h. The primary goal was to assess whether the available connection time—determined by the speed of the vehicle while passing through a reception zone—was enough to complete a full data exchange under realistic operational conditions. A positional layout can be seen in Figure 4.

It was assumed that the Data Mules usually traverse the area a sufficient number of times per shift and that no external interference affects communication. Hence, no further environmental factors were recorded because only topological characteristics have been shown to have any significance [9,21].

Key metrics include data delivery latency, the success rates of transmissions and the sizes of zones with less than 5% package loss, called reception zones per node. Distance route relates to the direct path that a vehicle can take or the direct path with the least open turns.

- Distance route to refueling station 1, T16: 280 m
- Distance route to refueling station 2, T3: 350 m
- Distance route to refueling server: 770 m
- Length of one Data Mule tour: 3500 m

### 2.3. Data Mule Concept

Data Mule describes a communication concept in which mobile machines or people are equipped with receivers to wirelessly collect data from sensors. The receivers then physically transport the data to a destination for upload. This approach is useful in scenarios where direct communication between nodes is not feasible or if the environment and conditions are subject to significant changes.

Data Mule networks can be used in a wide variety of configurations and environments. They are primarily ideal for situations in which there is low to no wireless connectivity between nodes for the planned data exchange. In addition, hardware requirements can often be reduced in the sensor and upload areas, as those points do not need to generate such strong radio signals to transmit to the remote node.

**Figure 5:**
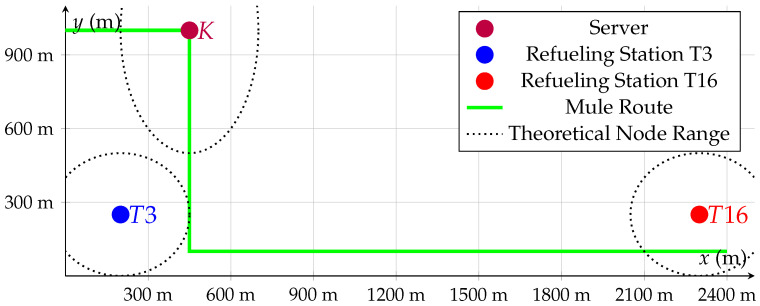
Labeled graph representation for Data Mule networks under given external restrains. Due to the influence of LoS conditions, the ranges for *K* are ellipsoid shaped, as it is placed in the drift and route of the Data Mule itself. Ranges are taken from experiments in [21].

Data Mule approaches and their algorithms represent a growing field in computer science [17,48,49,50,51]. Generally, these algorithms fall into the category of NP-hard problems known as the Traveling Salesman Problem with Neighborhoods [52]. These algorithms are based on fundamental parameters such as the number of agents (Data Mules), way-points, vertices, clusters of sensors, and trip durations. Even the feature that Data Mules can exchange data among themselves is not always considered part of the system [50,51,52]. A Data Mule network itself consists, both in planning and execution, of a series of individual algorithms that together enable the overall process. These include clustering algorithms that group sensors whose data can be collected in one trip, generative algorithms to optimize search through possible path trees, and more. In contrast to classical traveling salesman algorithms, these approaches add another dimension when considering temporal dependencies of data and their relation to real-time transmission. For example, k-Mule Scheduling [53] addresses determining the required number and duration of trips by Data Mules. Here, sensor capacity for storing and generating data must also be taken into account. In this application, the entire decision tree for scheduling could be brute-forced through a fixed set of external constraints. The basic planning algorithm was chosen specifically to demonstrate scalability to larger systems, since this setup—with only three key way-points—does not yet exceed algorithmic complexity limits. Critical to designing the Data Mule network were fixed parameters: two Jeeps (i.e., two Data Mules), three fixed way-points along a predetermined route defined by operational workflows, and realistically infinite data capacity and rate at both sensors and Data Mules (a few bytes per sensor reading—see Section 3). To visually illustrate how this Data Mule network can be mathematically analyzed in advance, a labeled graph representation will be used. This representation is also adopted as a method in [50]. This graph shows potential paths for the Data Mule on a 2D flat space. The set of *n* for the number of nodes, the active circular area of operation with radius *d* and the node to mule circular communication range of identical radius *r*, stands at the center of this plot—see Figure 5.

It should be noted that this study assumes a flat 2D Euclidean space for vehicle movement and signal propagation (see [50]), which is a common generalized assumption in such models. It is possible to extend Data Mule algorithms into the realm of curved metric spaces as well as into $R^{3}$ with a node range radius depending on all three space coordinates r(x,y,z). As distances are not equally dampened via the different LoS conditions, one would need to establish a circumvention. One way would be to redefine distances in respect to the signal budget cost. This would lead to a new norm with non-isotropic characteristics. Further information on norms can be found in [54,55].

Thus, only handshake algorithms and hardware implementation with radio options have remained open issues. The addition of Mule-to-Mule communication was a bonus feature demonstrating interoperability. In this use case, the Data Mule system can be implemented by upgrading existing mobile machines to collect data passively during regular tours related to use cases and specific requirements—see coverage ranges in Figure 5. These features allow Data Mule networks to react dynamically to changes in the environment. These changes can include shifts in the working area such as new vehicle routes or new operating points. Likewise, direct topological circumstances, such as new areas or rooms, often bring prearranged and fixed networks to a halt. In challenging environments, even with advance planning of transmission power and transmitter positions, these changes cannot be fully resolved without modifications to the network infrastructure itself. Many classic network structures or even mesh-based solutions are less flexible due to the need for a temporally uninterrupted connection duration for data exchange.

The core of the Data Mule principle is that a mobile unit can be set up to function both as a subscriber to data generation points such as sensors and as a publisher to upload to a database. Usually the following functionalities are integrated to increase the efficiency of the Data Mule approach: automation, exchange and synchronization. Automation means that the Data Mule vehicles immediately connect to data generators and servers without external command when a wireless connection is possible. They continuously try to establish it, since there is no timed guarantee of when the connection will be available. When the planned network has more than one Data Mule, server or sensor, the issue of data exchange becomes critical. The data must be sent via a communication option tailored to the environment and requirements so that the exchange remains secure and reliable. Short-range technologies like Bluetooth or NFC lack the signal budget for a reasonable area coverage. However, with the complexity of the data records and increased information, synchronization is necessary, which can take place on the Data Mule during transport, i.e., in a non-transmitting state. Synchronization can also be utilized for advanced systems between Data Mules themselves. Databases can be synchronized between sensors and Data Mules in order to transfer only new or missing data records, which reduces data volume and required connection up-time.

Several key evaluation parameters can be derived from these points:

A short overview of state-of-the-art implementations and use cases.

Ref. [56] is one of the foundational studies that have examined the concept in urban situations. The concept has been reevaluated over the years, for example in [57], where the main challenge to Data Mules of scheduling algorithms next to applications was discussed. This indicates the reason for the moderate level of research and the real-world application of the concept. After the concept itself was developed, the availability and cost-effectiveness of existing networks have enabled direct connections in most cases, reducing the necessity for alternative approaches. These studies give an overview of Data Mule algorithms like [58]. However, few studies have examined the extent to which non-urban environments profit from Data Mule networks, for example on the ocean floor [59]. Smart City solutions and other urban settings have been studied in more depth in publications like [18,60,61].

## 3. Hardware and Software Configuration

**Figure 6:**
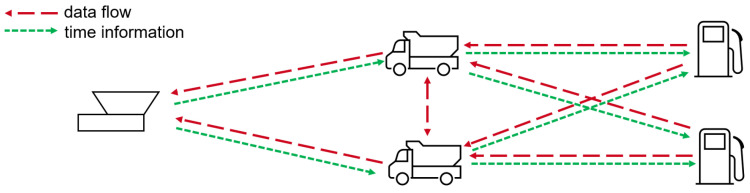
System topology and bidirectional flow of data and time synchronization information. Red dashed lines indicate the transfer of sensor data from the refueling stations to the Mules and onward to the server. Green dashed lines indicate the transmission of time synchronization information from the server to the Mules and then back to the refueling stations.

The Data Mule concept employs an intermediary node to establish a communication channel between entities that cannot directly exchange data. In this application, the system is designed to automatically transmit fuel-level information from distributed refueling stations to a control center. This section outlines the overall system architecture, followed by the hardware components. The information flow within the system and the software functionalities ensure reliable data transmission. Figure 6 illustrates the structural topology and bidirectional information flow.

As shown in Figure 6, the refueling station is located on the right, the Jeep—acting as the mobile Data Mule—in the center and the server, functioning as the control center, on the left. Sensor data is transmitted from the refueling station to the mule and subsequently to the server, ensuring the availability of up-to-date fuel-level information. In the opposite direction, time synchronization information is transmitted from the server to the Mule and then to the refueling stations. To realize this functionality, three types of LoRa-based hardware were developed for deployment at the refueling stations, on the Mules and at the server. All device types integrate a LoPy 4 module, combining an ESP32 micro-controller with an SX1276 LoRa transceiver, enabling both LoRa communication and on-board data processing.

At the refueling station, the hardware measures the remaining amount of fuel and transmits the data to the Mule. A VEGAPULS WL S 61 fuel-level sensor provides continuous monitoring, outputting a current signal in the range of 4 mA to 20 mA. The correlation between output current and fuel level is used to calculate the volumetric difference. A resistor is connected in series with the sensor’s output loop, and the voltage across it is measured to determine the output current. The micro-controller’s Analog-to-Digital Converter (ADC) provides this voltage. Both the micro-controller and LoRa module are powered by the station’s control cabinet. On the Jeep, the hardware receives data from the refueling station, stores it locally and forwards it to the server when in range. The system is powered by the Jeep’s onboard power supply.

At the server, a Raspberry Pi paired with an external LoRa module receives data from the Mule. This unit is powered by the station’s control cabinet.

With the hardware deployed at each site, communication between devices follows a broadcast-based mechanism. The LoRa modules at both the refueling station and the server continuously broadcast messages. At the refueling station, the first measurement in the queue is repeatedly transmitted until an acknowledgment is received. If no acknowledgment is received within a specified timeout, the message is retransmitted. Upon acknowledgment, the entry is removed from the queue and the next measurement is sent.

**Figure 7:**
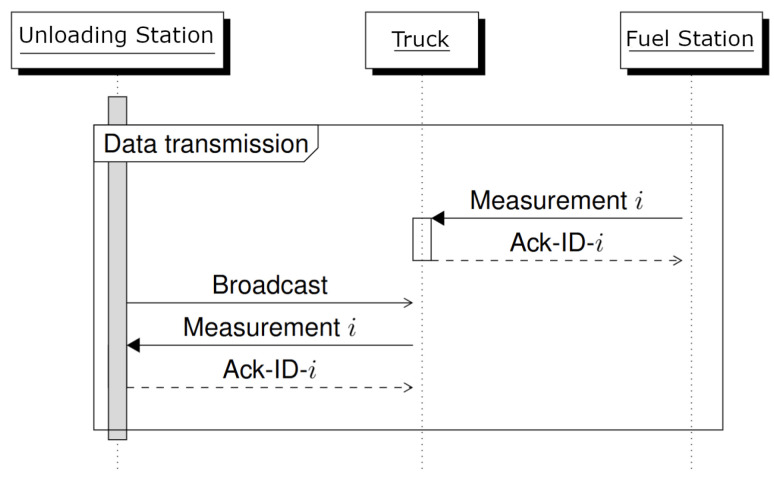
Data transmission sequence between system components. The server initiates communication by sending a broadcast request. The Mule relays this request to the refueling station, which returns measurement data *i* and its associated ID. Both the Mule and server send back acknowledgment messages (Ack-ID-*i*), which also carry time synchronization information.

The server continuously sends a beacon message. When a Mule receives this message, it indicates that it is within communication range. The Mule then transmits stored measurement data received earlier from the refueling station. After each acknowledged transmission the Mule proceeds to the next measurement. If three consecutive transmissions fail, the Mule enters silent mode, assuming it has left the communication range of the server—see Figure 7.

The software architecture focuses on the timely and efficient transmission of the most recent fuel-level measurements. To achieve reliable data transfer several software-level mechanisms were implemented, including message-type classification, acknowledgment-based delivery, time synchronization, queue management and event-based transmission.

To enhance scalability, messages are categorized by type. In the present system “Measurement” messages are used containing the message type, remaining fuel capacity and a unique message ID. The message ID consists of the device ID, device level and timestamp. Device levels define the communication hierarchy and permitted message flow: level 3 for refueling stations, level 2 for Mules and level 1 for the server. Measurement messages are transmitted only toward decreasing device levels. The receiving device sends a confirmation message of a designated acknowledgment type, ensuring reliable delivery.

Each confirmation message includes the message ID and embedded time information from the sending device’s system clock. While measurement data flows from higher to lower device levels, time synchronization flows in the opposite direction. This bidirectional exchange maintains clock accuracy across devices during long-term operation.

A Last-In-First-Out (LIFO) queue is used at the refueling station to prioritize the transmission of the most recent measurements. The queue stores up to 20 entries and discarded older data points.

An event- and time-based transmission strategy is implemented to improve efficiency. The micro-controller samples the fuel level every minute, and if the change from the most recent queued value exceeds a predefined threshold, the new value is added to the queue. Additionally, a heartbeat mechanism allows new measurements to be queued regardless of the change magnitude if the time since the last transmission exceeds a set limit. This approach reduces transmitted data, while ensuring timely updates compared to polling-based methods.

## 4. Results

Now follows the section describing and explaining the results from the measurement series. The core of this presentation consists of maps of the sub-areas, which are used to display and describe the signal paths, targets, and nodes.

The Data Mule network was deployed across two sections of the Werra potash mine at a depth of 800 m.

Refueling station T16 was located in the first section, to the east. The selected route between the two sections followed a straight drift without LoS, running orthogonally to a drift in the second section. The first drift was approximately 280 m long, and the second extended to about 350 m.

In the second section, refueling station T3 and the tipping point in the north were integrated as server nodes into the LoRa Data Mule network.

T16 and T3 were located off the main Data Mule routes in separate drifts.

Additionally, both T16 and T3 were not positioned directly within the center of their drifts but to the side, allowing larger vehicles such as Load-Haul-Dump (LHD) machines to pass through the drift.

Connectivity to T16, T3 and the server was possible at all tested speeds between 20 and 40 km/h.

Connections could be established both to the west and east of the drift leading to T16. In the western direction the range reached approximately 8–9 pillars (about 540 m), while in the eastern direction it was asymmetrical, reaching about two pillars (about 120 m). No gradual packet losses were recorded during the measurement period.

**Figure 8:**
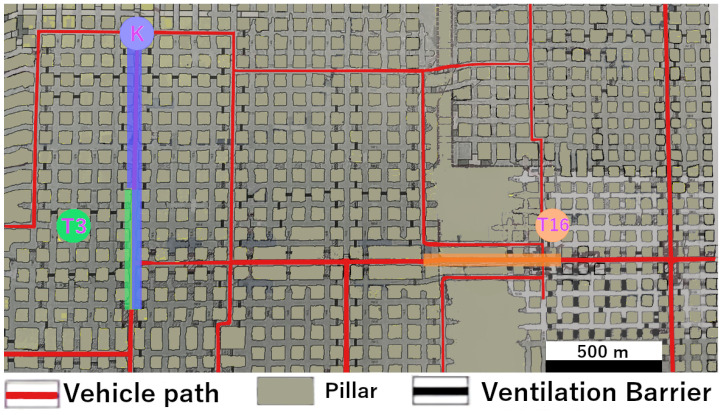
Excerpt from a map of the K+S mine near Werra, Germany. Points of interest like refueling stations and server areas are highlighted. T3 and T16 are refueling stations and K is the on site label for the tipping point, expanded with the server unit. Zones with LoRa network connectivity are color coded.

Key evaluation parameters revisited from Table 2:

Connections were established and lost abruptly but consistently within the boundaries shown in Figure 8.

The connection to T3 showed a similar pattern, covering both the southern and northern directions. The coverage here was more symmetrical, extending around four pillars (approximately 280 m) in each direction.

The tipping point was located within the center of the drift, allowing the server to be powered by a distribution box.

Measured coverage of the server extended over 14 pillars along the straight drift without LoS. All nodes here were based on the same LoRa hardware and operated at the same power level.

## 5. Discussion

In the following section, the results will be evaluated in relation to the problem statement presented in the introduction, with particular focus on the limitations regarding applications to similar use cases.

The primary objective was to eliminate manual collection of data and detouring of vehicles to fixed access points (e.g., refueling stations). The solution involved a fully passive Data Mule system using upgraded Jeeps. This objective was achieved. The Mules were able to collect data during regular tours and upload it to the central server. Furthermore, inter-vehicle data exchange was successfully recorded. It became evident that a Data Mule approach is particularly suitable for this use case. Even with LoRa providing long-range communication in underground environments, full-area coverage would require extensive infrastructure to achieve permanent connectivity.

Our measurements confirmed the non-linear nature of LoRa signal propagation. The near-LoS conditions in two dimensions reproduced the results of [21]. This study used model-supported feasibility planning rather than full model validation. The current propagation models remain insufficiently accurate for robust layout optimization, hence further model validation is required. Nonetheless, asymmetry was observed between T3 and T16, which is likely due to small but relevant topological differences in wall inclination. Even a difference of a few meters can significantly affect signal behavior.

As a result, coverage estimation is possible, but fully reliable predictions for signal path loss remain out of reach at this stage.

From a technical perspective, the LoRa Data Mule configuration posed no operational difficulties for the vehicles, even at varying speeds. Therefore, upgrading additional machines can increase network density and reliability. It should be noted that all vehicles, except for emergency vehicles during rescue missions, are limited to 40 km/h.

Vehicle-to-vehicle communication represents a valuable upgrade to the basic Data Mule system architecture. While not essential for the success of this experiment, it proved effective—especially when vehicles were in close proximity. For more complex scenarios or distributed node configurations, this functionality could provide a solution for temporarily bridging disconnected segments. Thus, it is a feature worth considering for future studies.

The results indicate that the Data Mule approach can be a viable solution and, when combined with LoRa, can cover wide and structurally complex environments of underground mines—see Table 3. However, any specific use case needs to be comparable to the one described/implemented here and be suitable for increasing operational efficiency. Data collection must be categorized as part of a real-time or right-time schedule, as discussed in [1].

For example, for refueling stations, right time means that a final count update is given at the end of the shift, which is acceptable and achievable. In contrast, critical information—such as a sudden increase in gas concentration—cannot be reliably or promptly communicated via a Data Mule concept.

Machines, operational states of ventilation systems, or fixed sensor units monitoring underground climate conditions are ideal candidates for Data Mule networks.

However, all relevant data points must be regularly reached or passed by vehicles. If certain locations are not covered frequently, a passive Data Mule approach will not suffice. In such cases, network relays or active route adjustments for vehicles should be considered.

An extension that was tested in this study is Mule-to-Mule communication. This feature can serve as an effective upgrade, particularly in minimizing coverage gaps by enabling connections between remote mining areas.

## 6. Conclusions

This paper has presented and demonstrated a LoRa-based Data Mule approach. The objective was to integrate several refueling stations at an active mining site operated by the K+S Group in Werra, Germany. They formed a network and server infrastructure in such a way that manual data retrieval at these stations would no longer be necessary.

The investigated area consisted of two mining districts and covered approximately 3.6 km^2^. Jeeps were equipped with Data Mule nodes and a central server was installed at a tipping point, along with nodes at two refueling stations.

Again, all these points are meant to be repositioned because they need to move with the mining front for supply and safety.

The results—see Table 3—demonstrate that LoRa Data Mules in an active potash mine environment are beneficial. Further verification of upgrades and improved scheduling are necessary to double or triple the network size. In such cases, k-Mule interactions [53] could impose a higher verification load between the Mules than was observed in this setup. Therefore, new software, including data processing for large-scale projects, needs to be developed. The current system can be expanded in this way.

These results demonstrate that Data Mule-based measurements are feasible, even though wave propagation still requires deeper understanding and improved modeling. Existing models, such as those described in [9,21], provide approaches for underground mining signal propagation but are not yet sufficiently robust. They lack the accuracy to enable reliable and optimized node layouts.

## Figures and Tables

**Table 1:** Selected network options compared. Qualitative comparison (− low/poor, o medium, + high/good).

Criteria	Wired Monitoring	Wi-Fi/5G	Direct LoRa Static Nodes	Data Mule with LoRa
Req. Infrastructure	−	−	o	+
Intrusiveness	−	o	o	+
Scalability	−	−	o	+
Network Performance	+	+	o	−
NLoS Practicality	o	−	o	+
Sources	[12,42,43,44]	[13,42,43,45]	[16,21,46,47]	[22,46,47]

**Table 2:** Key performance indicators and parameters for Data Mule network operation.

Metric	Reasoning
Package Loss	Below 5% PL can be recovered without issue and can guarantee secure, reliable data storage.
Travel Speed Jeep	Jeeps travel between 20 and 40 km/h; they should not slow down to receive relevant data.
LoS to the Emitter	LoS cannot be guaranteed underground; hence, it must operate reasonably without it.
Signal Range over 100 m	Significant areas of communication are at least 100 m apart.
Data Integrity	No false positive or positive false data packages must be stored after synchronization and verification on the Data Mule.
Hardware & Software	Hardware and software must not replace current installations. Changes due to tests must be reversible.
Other Networks	No LoRa influences on Wi-Fi or digital radio communications.
Training for Staff	Staff should not be involved in operation of the network sensor readout.

**Table 3:** Results for the key performance indicators of the network operation.

Metric	Result
Package Loss	Target < 5%; observed criterion met; 100% complete updates on the Data Mule servers.
Travel Speed Jeep	Recorded Jeep speeds at 20 to 40 km/h; No missed data packages. No Mule-to-Mule communication issues observed. No higher speeds could be tested due to general safety regulations. Mule-To-Mule communication and Galilean transformation indicates high-speed operation [62].
LoS to the Emitter	No LoS prior to successful connection. T16 and T3 were in different drifts to the travel route. The server positioned at K did not have an LoS for 300 m due to elevation changes.
Signal Range over 100 m	Transmission over 500 m with no direct LoS observed. See Figure 8.
Data Integrity	Only verified true-positive results were recorded on the Data Mule. Ack-ID were checked for successful data transfers, see Section 3.
Hardware & Software	Any hardware installation and removal could be done on powered down stations and Jeeps. Network components did not replace components but worked in parallel. New software did not extend into preexisting hardware, it was contained on new LoRa infrastructure.
Other Networks	LoRa was only installed in non Wi-Fi areas and channels differed by several GHz (Sub-GHz vs. 2.4 GHz and 5 GHz). No issues with the digital radio operation were recorded or reported.
Training for Staff	Active participation of staff happened during the installation. Operation was autonomous during regular tours and did not require actions by the personnel.

## Data Availability

The original contributions presented in this study are included in the article. Further inquiries can be directed to the corresponding author.

## References

[1] Clausen E., Sörensen A.M.A., Uth F.D., Lehnen F., Schwarze B., Mitra R. (2020). Assessment of the Effects of Global Digitalization Trends on Sustainability in Mining: Part I: Digitalization Processes in the Mining Industry in the Context of Sustainability.

[2] Theissen M., Kern L., Hartmann T., Clausen E. (2023). Use-Case-Oriented Evaluation of Wireless Communication Technologies for Advanced Underground Mining Operations. Sensors.

[3] Clausen E., Sörensen A.M.A., Uth F.D., Lehnen F., Schwarze B., Mitra R. (2020). Assessment of the Effects of Global Digitalization Trends on Sustainability in Mining. Part II: Evaluation of Digitalization Trends and Their Effects on Sustainability in the Global Mining Sector.

[4] Barnewold L., Lottermoser B.G. (2020). Identification of digital technologies and digitalisation trends in the mining industry. Int. J. Min. Sci. Technol..

[5] Zhou C., Damiano N., Whisner B., Reyes M. (2017). Industrial Internet of Things (IIoT) applications in underground coal mines. Min. Eng..

[6] Salam A. (2020). Internet of Things for Sustainable Community Development: Introduction and Overview. Internet of Things for Sustainable Community Development.

[7] Darling P. (2011). SME Mining Engineering Handbook.

[8] Ranjan A., Sahu H.B., Misra P. (2016). Wireless Sensor Networks: An Emerging Solution for Underground Mines. Int. J. Appl. Evol. Comput..

[9] Branch P. Propagation Measurements and Models of 915 MHz LoRa Radio in a Block Cave Gold Mine. Proceedings of the 2021 International Conference on Information Networking (ICOIN).

[10] Ranjan A., Sahu H., Misra P. (2020). Modeling and measurements for wireless communication networks in underground mine environments. Measurement.

[11] Leinonen M.E., Hovinen V., Vuohtoniemi R., Pärssinen A. 5G Radio Channel Characterization in an Underground Mining Environment. Proceedings of the 2024 18th European Conference on Antennas and Propagation (EuCAP).

[12] Zrelli A., Ezzedine T. (2018). Design of optical and wireless sensors for underground mining monitoring system. Optik.

[13] Zhan P. (2023). Application of 5G Communication Technology Based on Intelligent Sensor Network in Coal Mining. J. Sens..

[14] Li M., Liu Y. (2009). Underground coal mine monitoring with wireless sensor networks. ACM Trans. Sen. Netw..

[15] Zhao H., Yang W. (2018). An emergency rescue communication system and environmental monitoring subsystem for underground coal mine based on wireless mesh network. Int. J. Distrib. Sens. Netw..

[16] Bo L., Liu Y., Zhang Z., Zhu D., Wang Y. (2022). Research on an Online Monitoring System for Efficient and Accurate Monitoring of Mine Water. IEEE Access.

[17] Chehbour F., Doukha Z., Moussaoui S., Guerroumi M. Opportunistic Data Mules for Short Delay Smart City Applications. Proceedings of the 2018 International Conference on Smart Communications in Network Technologies (SaCoNeT).

[18] Zichichi M., Serena L., Ferretti S., D’Angelo G. DLT-based Data Mules for Smart Territories. Proceedings of the 2022 International Conference on Computer Communications and Networks (ICCCN).

[19] Press Kit-Nexgen SIMS—Nexgensims.eu. https://www.nexgensims.eu/press-kit/.

[20] Sridharan S., Fulga S. (2020). LoRa^®^ Connectivity Made Smarter with Low-Power, Front-End Modules.

[21] Theissen M., Kianfar A., Clausen E. (2025). LoRa Propagation and Coverage Measurements in Underground Potash Salt Room-and-Pillar Mines. Sensors.

[22] Krichler T., Mischo H., Sobczyk M. (2022). Untersuchungen zur Echtzeitbetriebsüberwachung im Untertägigen Bergbau.

[23] Ericsson How to Empower Smart Oil and Gas with Private 5G. https://www.ericsson.com/en/blog/2025/4/how-private-5g-empowers-smart-operations-oil-gas-industry.

[24] Sugumaran S., Sowmya J.M., Siva M.V., Rishitha N., Sagar M.K.G.S., Ganesh M. IOT Enabled Smart Fuel Station Management System. Proceedings of the 2024 2nd International Conference on Networking and Communications (ICNWC).

[25] Gu W., Song Y., Zhang Z., Zheng M. (2025). Innovative Research on the Interconnection of C-V2X Technology and Hydrogen Refueling Stations. Energy Eng..

[26] Ting Y.T., Chan K.Y. (2024). Optimising performances of LoRa based IoT enabled wireless sensor network for smart agriculture. J. Agric. Food Res..

[27] Ahmed M.A., Gallardo J.L., Zuniga M.D., Pedraza M.A., Carvajal G., Jara N., Carvajal R. (2022). LoRa Based IoT Platform for Remote Monitoring of Large-Scale Agriculture Farms in Chile. Sensors.

[28] Bakshi S.C., Roy G.C., Saicharan E., Parvathi E. On Underground Mine Communication Systems. Proceedings of the 26th International Conference on Distributed Computing and Networking.

[29] Rajani J. (2023). 5G and Wi-Fi Performance in Underground Mining. Master’s Thesis.

[30] Hennen E., Pekarski A., Storoschewich V., Clausen E. (2025). Stereo Vision-Based Underground Muck Pile Detection for Autonomous LHD Bucket Loading. Sensors.

[31] Škiljo M., Blažević Z., Dujić-Rodić L., Perković T., Šolić P. (2022). Self-Sensing Antenna for Soil Moisture: Beacon Approach. Sensors.

[32] Rappaport T.S. (2002). Wireless Communications: Principles and Practice.

[33] Phillips C., Sicker D., Grunwald D. (2013). A Survey of Wireless Path Loss Prediction and Coverage Mapping Methods. IEEE Commun. Surv. Tutor..

[34] Edirisinghe S., Galagedarage O., Dias I., Ranaweera C. (2023). Recent Development of Emerging Indoor Wireless Networks towards 6G. Network.

[35] Ozdenizci B., Coskun V., Ok K. (2015). NFC Internal: An Indoor Navigation System. Sensors.

[36] Mekki K., Bajic E., Chaxel F., Meyer F. Overview of Cellular LPWAN Technologies for IoT Deployment: Sigfox, LoRaWAN, and NB-IoT. Proceedings of the 2018 IEEE International Conference on Pervasive Computing and Communications Workshops (PerCom Workshops).

[37] Ikeda H., Kawamura Y., Tungol Z.P.L., Moridi M.A., Jang H. (2019). Implementation and Verification of a Wi-Fi Ad Hoc Communication System in an Underground Mine Environment. J. Min. Sci..

[38] Amulya B., Bhargavi D., Ankita N., Lavan R.G. (2024). Research on An IOT Based Smart E-Fuel Station using ESP-32. Int. J. Res. Appl. Sci. Eng. Technol..

[39] Turuk V.E., Verba V.S., Golovanova M.V., Golubtsov P.E., Evsikov M.V., Neronskiy L.B., Zaitsev S.E., Tolstov E.F. (2017). Strizh SAR for small Condor-E satellites. Sovrem. Probl. Distantsionnogo Zondirovaniya Zemli Iz Kosmosa.

[40] Shaengchart Y., Kraiwanit T. (2023). Starlink satellite project impact on the Internet provider service in emerging economies. Res. Glob..

[41] Testouri M., Elghazaly G., Hawlader F., Frank R. (2025). 5G-Enabled Teleoperated Driving: An Experimental Evaluation. arXiv.

[42] Kianfar A.E., Sherikar M., Gilerson A., Skora M., Stankiewicz K., Mitra R., Clausen E. (2022). Designing a Monitoring System to Observe the Innovative Single-Wire and Wireless Energy Transmitting Systems in Explosive Areas of Underground Mines. Energies.

[43] Li K., Yang J., Li Z., Xia W., Yi J. Intelligent Mining Monitoring and Early Warning System Based on Kalman Filter. Proceedings of the 2025 44th Chinese Control Conference (CCC).

[44] Einicke G.A., Duff E.S., Reid D.C., Ralston J., Cunningham J., Hainsworth D., Roberts J.M., Corke P. (2010). The Application of Wireless LANS in Mine Automation. https://www.semanticscholar.org/paper/The-Application-of-wireless-LANS-in-mine-automation-Einicke-Duff/f263eae225ee261f53e80c811bdbc31919ac3409.

[45] Hasan S., Sjöberg K., Wallin P. (2025). Wireless Communication in Underground Mining Teleoperation: A Systematic Review. IEEE Access.

[46] Yadav D.K., Mishra P., Jayanthu S., Das S.K. (2022). On the Application of IoT: Slope Monitoring System for Open-cast Mines Based on LoRa Wireless Communication. Arab. J. Sci. Eng..

[47] Khan M.A.A., Luo S. (2026). Dual resource allocation for enhancing vehicle tracking using deep neural network and Bayesian LoRa. Expert Syst. Appl..

[48] Bonola M., Bracciale L., Loreti P., Amici R., Rabuffi A., Bianchi G. (2016). Opportunistic communication in smart city: Experimental insight with small-scale taxi fleets as data carriers. Ad Hoc Netw..

[49] Luo Y., Zhu X., Long J. (2019). Data Collection Through Mobile Vehicles in Edge Network of Smart City. IEEE Access.

[50] Sugihara R., Gupta R.K. (2011). Path Planning of Data Mules in Sensor Networks. ACM Trans. Sens. Netw..

[51] Hu Y., Zhang F., Tian T., Ma D., Shi Z. (2022). Shortest path planning of a data mule in wireless sensor networks. Wirel. Netw..

[52] Liu J.S., Wu S.Y., Chiu K.M. Path planning of a data mule in wireless sensor network using an improved implementation of clustering-based genetic algorithm. Proceedings of the 2013 IEEE Symposium on Computational Intelligence in Control and Automation (CICA).

[53] Citovsky G., Gao J., Mitchell J.S.B., Zeng J., Bose P., Gąsieniec L.A., Römer K., Wattenhofer R. (2015). Exact and Approximation Algorithms for Data Mule Scheduling in a Sensor Network. Algorithms for Sensor Systems.

[54] Rudin W. (2013). Real and Complex Analysis.

[55] Hieber M. (2019). Analysis II.

[56] Pentland A., Fletcher R., Hasson A. (2004). DakNet: Rethinking connectivity in developing nations. Computer.

[57] Bose P., Gąsieniec L.A., Römer K., Wattenhofer R. (2015). Algorithms for Sensor Systems: 11th International Symposium on Algorithms and Experiments for Wireless Sensor Networks, ALGOSENSORS 2015, Patras, Greece, 17–18 September 2015, Revised Selected Papers.

[58] Chang C.Y., Lin C.Y., Hsieh C.Y., Ho Y.J. Patrolling Mechanisms for Disconnected Targets in Wireless Mobile Data Mules Networks. Proceedings of the 2011 International Conference on Parallel Processing.

[59] Weng Y., Chun S., Sekimori Y., Yokohata H., Matsuda T., Pajarinen J., Maki T. (2025). Reinforcement learning-based tracking control of autonomous underwater vehicles for seafloor platform data collection. Ocean. Eng..

[60] Chehbour F., Doukha Z., Moussaoui S., Guerroumi M. (2020). OMS: Opportunistic mules for short latency data collection in smart cities. Int. J. Commun. Syst..

[61] Lan K., Wu Z.M. On the feasibility of using public transport as data mules for traffic monitoring. Proceedings of the 2008 IEEE Intelligent Vehicles Symposium.

[62] Arnold V.I. (2010). Mathematical Methods of Classical Mechanics.

